# ASO Author Reflections: The Value of Serum CEA for Prognostication at Staging and Response Evaluation in Patients with Localized Pancreatic Adenocarcinoma and Nonelevated CA19-9

**DOI:** 10.1245/s10434-023-14815-3

**Published:** 2024-01-05

**Authors:** Thomas F. Stoop, Deesje Doppenberg, Matthew H. G. Katz, Ching-Wei D. Tzeng, Alice C. Wei, Amer H. Zureikat, Bas Groot Koerkamp, Marc G. Besselink, for the Trans-Atlantic Pancreatic Surgery (TAPS) Consortium, Susan van Dieren, Rutger T. Theijse, Quisette P. Janssen, Naaz Nasar, Laura R. Prakash

**Affiliations:** 1 Amsterdam UMC, location University of Amsterdam, Department of Surgery, Amsterdam, the Netherlands; 2 Cancer Center Amsterdam, Amsterdam, the Netherlands; 3 Division of Surgical Oncology, Department of Surgery, University of Colorado Anschutz Medical Campus, Aurora, CO USA; 4 Department of Surgical Oncology, Division of Surgery, The University of Texas MD Anderson Cancer Center, Houston, TX USA; 5 Department of Surgery, Memorial Sloan Kettering Cancer Center, New York, NY USA; 6 Division of Surgical Oncology, University of Pittsburgh Medical Center, Pittsburgh, PA USA; 7 Department of Surgery, Erasmus University Medical Center, Rotterdam, the Netherlands

## Past

Disease staging and response evaluation following chemotherapy for localized pancreatic adenocarcinoma is based on anatomical, biological, and conditional parameters.^1^ Serum carbohydrate antigen 19-9 (CA19-9) is the only tumor marker recommended by guidelines for the purpose of biological (re)staging.^2^ However, approximately one-third of the patients have nonelevated serum CA19-9 at diagnosis,^3^ which hampers adequate response evaluation and clinical decision-making. Serum carcinoembryonic antigen (CEA) has been proposed as alternative biological tumor marker, but evidence about its prognostic value at time of diagnosis and restaging after chemotherapy as initial treatment is limited.^2^

## Present

The current, retrospective, multicenter study investigated the association of serum CEA with overall survival (OS) among 277 patients with localized pancreatic adenocarcinoma who were treated with (m)FOLFIRINOX as initial treatment, having nonelevated (i.e., <37 U/ml) serum CA19-9 at baseline.^4^ In this subgroup, serum CEA was elevated in one-third of patients. Nevertheless, both at baseline and at restaging, elevated serum CEA (as measured at baseline) was the only predictor for (worse) OS.

## Future

Serum CEA levels at baseline may be a useful tool for both decision-making at initial staging as well as at time of restaging in patients with nonelevated CA19-9. Future research should validate these findings and assess the prognostic value of serum CEA in patients with(out) elevated serum CA19-9, including the interaction and correlation between both tumor markers with their prognostic value. Moreover, alternative tumor markers are required as serum CEA at baseline is elevated in only one-third of patients having nonelevated CA19-9.^2,5^
